# 3. EXCITATION-INHIBITION IMBALANCES IN SCHIZOPHRENIA: MECHANISMS AND INTERVENTIONS

**DOI:** 10.1093/schbul/sby014.002

**Published:** 2018-04-01

**Authors:** Lawrence Kegeles

**Affiliations:** Columbia University & New York State Psychiatric Institute

## Abstract

**Overall Abstract:** Evidence is accumulating that core features of schizophrenia (SCH) may arise from a fundamental disturbance in the cellular balance of excitation and inhibition (E-I balance) within neural circuitry. In the symposium, we will provide a comprehensive overview of E-I balance alterations in SCH with evidence from preclinical models and in vivo measurements investigating potential neurobiological mechanisms underlying these dysfunctions as well as interventions that remedy these disturbances.

Takao Hensch will summarize findings on critical period plasticity and its potential role in vulnerability to schizophrenia. He will present new preclinical data on the destabilizing consequences of enhanced gamma oscillations, which reversibly prolong juvenile forms of brain plasticity by redox imbalance.

Jan-Harry Cabungcal will address the role of redox dysregulation and oxidative stress in the pathophysiology of schizophrenia. He will present recent data on the relationship of deficits of the perineuronal net and oxidative stress in the anterior cingulate cortex, and evidence that redox dysregulation can be targeted with antioxidants/redox regulators across animal models.

Lawrence Kegeles will present simultaneous EEG and proton MRS measurements of glutamate and GABA during ketamine administration in healthy young adults. These data will be compared with the same modalities acquired in individuals at clinical high risk and patients with SCH, showing disturbed delta and gamma band power and altered E-I balance despite homeostatic rebalancing of glutamate and GABA.

Peter Uhlhaas will summarize evidence from EEG/MEG data examining the potential role of neural oscillations in the pathophysiology of schizophrenia. He will show that alterations in gamma-band oscillations are present prior to the onset of schizophrenia in at-risk individuals and related to aberrant E-I balance parameters revealed by MRS-measured levels of GABA and glutamate. Developmental data on the maturation of neural oscillations suggests that the transition from adolescence to adulthood is a sensitive period for modifications in neuronal dynamics that could potentially explain the manifestation of psychosis during this period.

